# Cost-effectiveness of a bone substitute delivering gentamicin in the treatment of chronic osteomyelitis of long bones: Protocol for the CONVICTION randomized multicenter study

**DOI:** 10.3389/fmed.2023.1116711

**Published:** 2023-03-30

**Authors:** Hassan Serrier, Laure Huot, Sophie Brosset, Cécile Batailler, Tristan Ferry

**Affiliations:** ^1^Health Economic Evaluation Service, Hospices Civils de Lyon, Lyon, France; ^2^Université Claude Bernard Lyon 1, Research on Healthcare Performance RESHAPE, Lyon, France; ^3^Centre interrégional de référence pour la prise en charge des infections ostéoarticulaires complexes, CRIOAc Lyon, Hospices Civils de Lyon, Lyon, France; ^4^Department of Plastic Surgery, Croix-Rousse Hospital, Lyon University Hospital, Lyon, France; ^5^Department of Orthopaedic Surgery, Croix-Rousse Hospital, Lyon University Hospital, Lyon, France; ^6^Univ Lyon, Claude Bernard Lyon 1 University, Bron, France; ^7^Infectious Diseases, Hôpital de la Croix-Rousse, Hospices Civils de Lyon, Lyon, France; ^8^CIRI–Centre International de Recherche en Infectiologie, Inserm, Université́ Claude Bernard Lyon 1, Ecole Normale Supérieure de Lyon, Univ Lyon, Lyon, France; ^9^Université Claude Bernard Lyon 1, Lyon, France

**Keywords:** chronic osteomyelitis, osteoarticular infection, local antibiotic therapy, bone substitute, antibiotic resistance, cost-effectiveness

## Abstract

**Introduction:**

Chronic osteomyelitis is a serious osteoarticular infection that most often occurs in the long bones, responsible for significant morbidity with the risk of fracture and amputation. Despite advances in both antibiotics and surgical treatment, the probability of recurrence of infection remains at around 20%. Cerament-G (BONESUPPORT AB, Sweden) is a synthetic bone substitute that fills the bone void left by surgery, prevents infection and promotes bone regeneration within this space. Cerament-G also provides the local delivery of high doses of gentamicin over several weeks. Two prospective observational studies described a number of infectious recurrences of 4 and 5% after the use of Cerament-G. Although available in France, Cerament-G is currently not reimbursed and its high cost constitutes a barrier to its use. We hypothesize that the use of Cerament-G will lead to fewer costs to the collectivity while improving patient utility and, as an innovative strategy, will be superior to standard of care on recurrence of infection.

**Methods and analysis:**

The Conviction Study is a prospective, multicenter, randomized, single blind study conducted in 14 French Reference Centers for Complex Osteoarticular infections. The main objective is to evaluate the cost-effectiveness of using Cerament-G in the treatment of chronic long bone osteomyelitis by comparing this innovative strategy to standard of care. A cost-utility analysis from the collective perspective will be conducted over a 24-month time horizon after the initial surgery. The outcome for the main medico-economic evaluation will be Quality Adjusted Life Years (QALYs).

**Discussion:**

The study is being conducted throughout the CRIOAc network in France, in referral centers for the management of complex infections which will facilitate patient recruitment. This study has several limitations: the investigators have to be trained to handle the device, and it was impossible to blind the surgeon.

**Conclusion:**

If the use of Cerament-G is demonstrated to be superior to leaving the dead space empty during surgery for patients with stage III chronic long bone osteomyelitis, its use will be recommended to improve the prognosis of such patients, and this device may eventually qualify for reimbursement through the French Health Insurance scheme.

**Ethics and dissemination:**

This protocol received authorization from the Ethics Committee CPP Sud Méditerranée V on April 27, 2021 (21.03.10.77652) and the French National Agency for Medicines and Health Products on May 6, 2021 (2020-A02299-30). Results will be disseminated to the scientific community through congresses and publication in peer-reviewed journals.

## 1. Introduction

### 1.1. Rationale

Chronic osteomyelitis (stage III of the Cierny-Mader classification) is a serious osteoarticular infection which most often occurs in the long bones (tibia, femur, humerus, forearm), responsible for significant morbidity with risk of fracture and amputation. It is due to the presence of bacteria in the bone marrow, sometimes responsible for an intraosseous abscess, also called brodie abscess (1).

Chronic osteomyelitis can have a hematogenous or more often exogenous origin, after trauma or surgery. The bacteria involved have the ability to modify their metabolism and bring into play persistence mechanisms (such as biofilm) making their eradication difficult. The treatment of chronic osteomyelitis of long bones requires surgery, corticotomy: opening of the cortical bone to perform endomedular curettage to identify the bacteria, remove any sequestra (bone fragments to which the bacteria adhere in the form of biofilm), and reduce bacterial inoculum. Indeed, debridement of the intramedullary canal is essential to remove sequestrum where the bacteria is embedded in biofilm, to cure the disease. Access to the endomedular space involves the cutting of an elongated rectangular window parallel to the axis of the bone. This is the best approach structurally. The window should be no larger than 7 to 10 mm in diameter and 3 to 9 cm in length, depending on the size of the bone. If the size has to be bigger, the surgeon has to evaluate the risk of iatrogenic fracture and the potential need for preventive stabilization (usually not needed when ≥70% of the original cortex remains intact). Patients at particularly high risk of fracture (15% to 25%) may also need preventive stabilization during surgery (osteosynthesis or external fixator). The management of the “dead space” produced by endomedular debridement is still debated. In order to avoid the occurrence of post-debridement endomedular hematoma, some authors have discussed several possibilities. Some suggest autologous bone graft. Some prefer the use of antibiotic-impregnated PMMA cement; beads or cementoplasty that fill the entire bone void, but this strategy would require a new surgical intervention to remove the cement. There is also the Papineau technique involving serial open bone grafting. Lastly, there is the possibility of skin graft, whereas bone grafting is not biomechanically useful in stage III osteomyelitis. None of these techniques are considered as the standard of care procedure for bone void management, practices seem to be largely heterogeneous, and leaving the dead space empty, is clearly also an option to limit complications induced by iterative interventions. Indeed, a two-stage approach, with use of a PMMA cement to prepare the performance of a bone graft during the second stage, has to be considered during stage IV osteomyelitis (septic non-union), but not in stage III osteomyelitis. The two-stage approach would help to obtain an induced membrane that facilitates the bone grafting, and then obtain the consolidation of the disrupted long bone, i.e., to restore the continuity and the union of the long bone. Indeed, there is no biomechanical need for bone grafting in stage III osteomyelitis of long bone. However, in stage III as in stage IV osteomyelitis, a surgical act of “skin and soft tissue cover” called “flap” can be necessary, in particular in patients presenting an old attack with weakening and adhesion of the skin and soft tissues to the underlying bone. The flap could be done during the same time as the bone debridement, or could be done during a second operative procedure (2, 3).

Post-operatively, the patient receives a probabilistic systemic antibiotic therapy then a systemic antibiotic therapy targeted on the identified germ, for a period of 3 months. The effectiveness of these antibiotics relies on their ability to penetrate bone tissue. Despite the progress made in both antibiotics and surgical treatment, the probability of failure of this treatment (infectious recurrence) is around 20%, and has unfortunately remained stable for more than 20 years (1, 4). A single-center study published in 2018 (4) followed 116 patients for at least 1 year after discharge from hospital for treatment of osteomyelitis of the long bones of the lower and upper limbs (the class was not specified). In total, 26 infectious recurrences (22.4%) were observed with an average delay of 11.2 months (95% CI = 3.3–19.1). Recurrences could be explained by the persistence of the initial germ despite a well-conducted treatment or by superinfections with other bacteria, acquired during surgery, which took advantage of the “dead space” generated by the surgical curettage. In the event of failure, a new surgical and antibiotic sequence is often proposed, with a new risk of fracture or even amputation (approximately 5% of patients experienced treatment failure) due to bone weakening and iterative surgeries.

Local antibiotic therapy could be a solution, but so far, it has not been clearly considered and recommended due to the lack of a “carrier” or a device that could stabilize and release an antibiotic locally over several weeks.

Some bone substitutes have been developed in this indication, such as Osteoset-T (WRIGHT MEDICAL FRANCE, France), a synthetic bone substitute composed of calcium sulfate and supplemented with tobramycin, CE marked and marketed in Europe in 1998. It was approved by the French National Health Authority for bone filling on an infected site in cases of osteitis on continuous bone (5). A 2018 report by the Health Technology and Intervention Evaluation Unit of the University Hospital Centre of Québec, on the use of calcium sulfate impregnated with antibiotics for the prevention and treatment of infections, concluded that: “the available data suggest a possible beneficial effect of calcium sulfate impregnated with antibiotics in the treatment of infections and more particularly of osteomyelitis.” However, the literature review carried out showed great heterogeneity in the studies and practices used for the management of these patients, in the types of substitutes (components, types and doses of antibiotics), and in the definition of recurrence and follow-up periods. The publications selected studied Osteoset-T or Stimulan (calcium sulfate beads impregnated extemporaneously with an antibiotic), and reported an infection eradication rate of between 80 and 92% in comparative studies, with a minimum follow-up of less than 1 year post-surgery. The only randomized study (6) compared polymethyl methacrylate cement beads (non-absorbable) impregnated with an antibiotic with Osteoset-T. A total of 14 patients were included in each group, and 2 patients had an infectious recurrence in each group (14%; mean follow-up 38 months). In terms of tolerance, the main risks of adverse effects found in the literature, with a low occurrence rate, were related to calcium sulfate (hypercalcemia, inflammatory reactions with exudates around wounds of a serious nature, heterotopic ossification, and allergy) or depended on the type of antibiotic used, including a risk of transient acute renal failure. These data are of real interest for the management of a disease such as chronic osteomyelitis; unfortunately, Osteoset-T has not been available on the French market since 2010.

A new synthetic bone substitute composed of hydroxyapatite, calcium sulphate, and gentamicin, Cerament-G (BONESUPPORT AB Laboratory, Sweden), has been developed and was CE marked in 2013. This substitute fills the “dead space” that is formed during surgery, prevents this cavity filled with blood from becoming infected, and promotes the regeneration of the bone within this space, limiting the risk of fracture to medium and long term. The device comes as a powder to be reconstituted in a pre-filled syringe and takes the form of a paste that hardens over time. There is a precise timing to be observed, between the time of mixing, the application, and skin closure. Cerament-G also delivers locally for several weeks very high doses of gentamicin (concentration of 17.5 mg/ml in the device), a broad-spectrum bactericidal antibiotic effective against the vast majority of bacteria involved in osteoarticular infections. It provides effective local antibiotic therapy through broad local exposure to this antibiotic, at prolonged concentrations for several weeks. Initially around 1,000 mg/l, the local concentrations, thus obtained, then remain well above the minimum inhibitory concentration of the bacteria potentially involved, with local release of gentamicin for more than 21 days (7). Low plasma concentrations of gentamicin have been noted (8, 9) showing a low passage of the antibiotic systemically.

The combination of local and systemic antibiotic therapy is expected to provide a benefit in terms of anti-infective activity. The high local concentrations of gentamycin diffused in the hours and days following insertion by the device are complementary to systemic antibiotic therapy, which uses molecules whose bone penetration is variable. There is a real theoretical complementarity in terms of pharmacokinetics between local antibiotic therapy and systemic antibiotic therapy. The composition of Cerament-G (hydroxyapatite and calcium sulphate) also promotes bone regeneration and could therefore limit the risk of fracture following corticotomy.

A prospective study was conducted on 100 patients with chronic class III (*n* = 78) or IV (*n* = 22) osteomyelitis (105 bones) (10). The treatment was performed by one-step debridement surgery with the addition of Cerament-G, between March 2013 and February 2015, with a postoperative follow-up of 12 to 34 months (19.5 months on average). An infectious recurrence was observed in 4 patients between 145 days (approximately 5 months) and 563 days (approximately 18 months), all class III (i.e., 5%, 4/78). Recurrence was defined by a positive culture following a biopsy or an aspiration guided by radiology. Eleven patients presented with a minor extraosseous leakage of Cerament-G, visible on radiology, without consequences for the patients. In 6 cases, white wound drainage fluid, with the appearance of liquefied calcium sulphate residues, was observed.

A second prospective non-randomized monocentric study compared 3 antibiotic-impregnated bone substitutes as an adjunct to surgery for the treatment of patients with class III or IV chronic osteomyelitis (11): Septocoll-E (*n* = 74, mean follow-up 1.75 years); Osteoset-T (*n* = 166, mean follow-up 1.96 years); and Cerament-G (*n* = 73, mean follow-up 1.78 years—including 64 class III patients). The infectious recurrence rate for the group treated with Cerament-G was 4.1% (3 patients). In this group, the fracture rate was 1.4% and the leak rate 9.6%. These results were lower than those observed in the other groups (notably twice as low as in the Osteoset-T group; statistically significant differences), despite a percentage of patients presenting a higher risk of recurrence.

Cerament-G is a potential breakthrough innovation in the management of chronic osteomyelitis, but its use is limited in France due to its high cost (up to about €4,000 per procedure depending on the dose), which is not covered by the national health insurance.

However, its use as an adjuvant in the treatment of chronic osteomyelitis of the long bones is expected to avoid resource consumption such as the cost of managing failures (repeated surgeries, prolonged hospitalizations, new intravenous and then prolonged oral antibiotic therapies with sometimes expensive molecules, stays in follow-up care and rehabilitation, etc.) and the cost of managing possible fractures or amputations. The cost of a hospital stay for the treatment of an osteoarticular infection is high since it is estimated by the French national cost study at between €5,518 and €19,608 depending on the level of severity. Similarly, the cost of a hospital stay is estimated between €5,130€ and €18,481 for the amputation of a leg and between €1,875 and €8,892 for a leg fracture depending on the level of severity. At the same time, it will lead to an improvement in the quality of life of patients, in particular because it will make it possible to avoid painful and sometimes ineffective repeated surgeries that can lead to fractures or even amputations. In diabetic patients, Clarke et al. (12) estimated the decrease in utility associated with a limb amputation at −0.280 in the short term. The impact on the utility of a leg amputation is less important in the long term but would always be between −0.039 and −0.173 (13) depending on the treatment. Similarly, the disutility linked to a femur fracture has been estimated at −0.258 in the short term and between −0.058 and −0.402 in the long term depending on the management (13).

The French multicenter CONVICTION study has been developed based on these assumptions, to assess the efficiency of the use of Cerament-G as an adjuvant in the treatment of chronic osteomyelitis of the long bones.

### 1.2. Objective and hypothesis

The main objective is to evaluate the cost-effectiveness of the use of Cerament-G in the treatment of chronic osteomyelitis of long bones. Two strategies will be compared: medico-surgical usual care based on corticotomy (± skin and soft-tissue/muscle flap) followed by systemic antibiotic therapy (standard of care); and medico-surgical care with corticotomy during which Cerament-G is used, followed by systemic antibiotic therapy (innovative strategy).

We hypothesize that, despite the initial additional cost associated with Cerament-G, its use will lead to fewer future costs while improving patient utility. We also hypothesize that the innovative strategy is superior to the standard of care in terms of recurrence of infection.

This study will focus on adult patients with chronic osteomyelitis of long bones, requiring surgical management in one of the referral centers for the management of complex osteoarticular infections in France (“Centre de Référence des Infections Ostéo-Articulaires complexes” [CRIOAc]).

## 2. Methods and analysis

### 2.1. General information

The trial has been registered at the Clinical Trials Registry as NCT04805164.

The CONVICTION Study is a prospective, multicenter, randomized, single blind study comparing two treatment strategies for chronic osteomyelitis of long bones with and without the use of Cerament-G.

Patients will be recruited as part of management care of their chronic osteomyelitis in a CRIOAc, and will be enrolled by the investigator after evaluation in a multidisciplinary consultation meeting.

The participating centers are orthopedic surgery and infectious disease departments from 12 French University Hospital Centers. Study sites can be obtained from the Sponsor’s representative.

Four committees were created as part of this study: a Scientific Committee; a Patient Eligibility Validation Committee; a Clinical Event Validation Committee; and an Independent Safety Committee.

The role of the Scientific Committee is to ensure that the study runs smoothly. The Scientific Committee also participated in the drafting of the protocol. The committee will be regularly informed of the progress of the study by the coordination and, if necessary, will be called upon to make decisions on the study protocol.

The members of the Patient Eligibility Validation Committee will ensure that each patient has a relevant indication for Cerament-G, based on clinical and radiological data. They will propose direct closure without tension or a cover flap following the initial surgery, and the addition of a possible preventive stabilization in case of high risk of fracture (internal fixation or external fixator). Finally, they will specify the volume of Cerament-G to be injected.

The Clinical Event Validation Committee will be composed of three members independent from participating centers, with the task of reviewing available data from each case to comment on the nature of the clinical events (healing, remodeling/consolidation and recurrence of infection) measured by the investigators. The members of this committee will be blinded to patient treatment.

The Independent Safety Committee will only meet if more than three recurrences of infection are observed in the innovative strategy group during the first year of the study. Its members will have to give an opinion on whether or not to continue the study, based on all relevant data, including the conclusions of the Clinical Event Validation Committee.

### 2.2. Participants

For the duration of the study, the Sponsor has taken out insurance covering its own civil liability as well as that of any doctor involved in carrying out the study. He will also ensure full compensation for the harmful consequences of research for the person who lends himself to it and his beneficiaries, unless he can prove that the damage is not attributable to his fault or to that of any intervening party, without that may be opposed the act of a third party or the voluntary withdrawal of the person who had initially consented to participate in the research.

#### 2.2.1. Inclusion criteria

- Patient with suspected chronic osteomyelitis (stage III of the Cierny-Mader classification) of a long bone of the tibia, femur, humerus or forearm, at the diaphysis, metaphysis or epiphysis, defined as follows:
  o Supposed inoculation >3 months;
  o At least one of the following clinical signs at the suspected infected site:
  ▪ Spontaneous or bearable pain while walking;
  ▪ Presence of a fistula or history of leakage through a fistula;
  ▪ Presence of serous or purulent flow;
  ▪ Presence of bone exposure;
  ▪ Local inflammation;
  ▪ Fever in the absence of any other explanation.
  o At least one of the following radiological signs at the suspected infected site:
  o Bone reshaping with osteolysis or periosteal apposition;
  o Presence of intramedullary abscess (if Magnetic Resonance Imaging [MRI] performed);
  o Presence of a fistulous pathway to the intramedullary (if MRI performed);
  o Presence of bone sequestration visible on CT scan (if CT scan performed).
- Patient eligible for conventional surgical treatment of chronic osteomyelitis, with decortication and corticotomy with endomedular debridement (to eradicate bone sequestrums, reduce the inoculum, and identify the bacterium(s) involved) and with secondary intramedullary residual cavity that does not need cementoplasty (osteomyelitis that needs induced membrane technique and cementoplasty are to be considered as stage IV osteomyelitis);
- Patient having 3 months of systemic antibiotic therapy post-operatively planned;
- If osteosynthetic material is present in the infection site, this material should be considered preoperatively as completely removable during chronic osteomyelitis surgery;
- Patient eligible for a direct closure without tension, or eligible for skin and soft-tissue/muscle flap to be performed within 15 days after initial surgery;
- Male or female patient between 18 and 80 years of age;
- Patient who has given written informed consent to participate in the study;
- Geographically stable patient;
- Patient able to comply with follow-up visits, protocol schedule and therapeutic treatment, according to investigator’s judgement;
- Patient affiliated to or benefiting from a social security system.

#### 2.2.2. Exclusion criteria

- Acute hematogenic osteomyelitis (Cierny-Mader stage I);
- Cortical osteitis (Cierny-Mader stage II);
- Septic pseudoarthrosis (Cierny-Mader stage IV);
- Patient requiring an estimated skin and soft-tissue/muscle flap that cannot be done within 15 days after surgery for the treatment of chronic osteomyelitis;
- Female who is pregnant, nursing or who is considering becoming pregnant during the study period;
- Patient participating in another interventional study
- Patient known to have hypersensitivity to aminoglycosides (especially gentamicin), sulfites (including calcium sulphate) or calcium hydroxyapatite;
- Contraindication to the use of Cerament-G: severe myasthenia (class IV or higher according to the MGFA classification), severe renal insufficiency (creatinine clearance <30 ml/min according to the Cockcroft-Gault formula, or GFR < 30 ml/min/1.73m^2^ according to the CKD-EPI or MDRDs equation or, dialysis patient), pre-existing disorders of calcium metabolism (total plasma calcium (or total corrected plasma calcium according to albuminemia) outside normal laboratory values);
- Patient with endocrine or metabolic disorders known to affect osteogenesis (e.g., Paget’s disease, renal osteodystrophy, hyperthyroidism, parathyroid disorder, Ehler-Danlos syndrome, osteogenesis imperfecta);
- Patient with one or more untreated malignant cancers (including Marjolin’s ulcer), or undergoing radiotherapy or chemotherapy;
- Adult patient protected by law, under guardianship or trusteeship.

#### 2.2.3. Criteria for early withdrawal from the study

- Invalidation of the patient’s inclusion by the Patient Eligibility Validation Committee;
- Deprogramming and definitive cancelation of surgery for the treatment of chronic osteomyelitis;
- Discovery (between the validation of the inclusion and the surgery) of the inability to perform the soft-tissue/muscle flap within 15 days after the initial surgery;
- Discovery of hypercalcemia or unknown severe renal failure between the inclusion visit and surgery.

### 2.3. Randomization

Patients will be randomized between the two strategies in a balanced manner with a computerized and secure system *via* the internet. Randomization will be carried out by minimization, using a computer algorithm that calculates in real time the allocation of the group that guarantees the best possible balance, taking into account the patient’s stratification values (center and preventive stabilization) as well as the patients already randomized. So that the randomization is not predictable, a part of randomness will be added to the algorithm.

### 2.4. Experimental design

#### 2.4.1. Study treatments

Cerament-G is an absorbable ceramic bone substitute composed of calcium sulfate, hydroxyapatite, and gentamicin sulfate, intended to fill bone gaps and deficits in the skeleton and promote bone healing. This is a medical device with CE marking since February 11, 2013, and manufactured by the Bonesupport laboratory (Lund, Sweden). The product, supplied in 5 ml or 10 ml format, consists of a powder and a liquid component. The powder contains 40% hydroxyapatite and 60% calcium sulfate hemihydrate. The liquid component is saline solution and gentamicin. The mixture of the components gives a viscous material suitable for direct or percutaneous injection into a bone void. By combining hydroxyapatite and calcium sulfate, an optimal balance is achieved between the rate of implant resorption and the rate of growth in bone. Calcium sulfate acts as an absorbable carrier for hydroxyapatite. Hydroxyapatite, a substance with a low resorption rate and a high osteoconductive profile that promotes bone growth, provides long-term structural support for new bone tissue. The hardened material facilitates bone growth and based on the observation of serial x-rays in a large number of patients and various conditions, it appears to be replaced by bone over a period of 6 to 12 months.

Cerament-G mixed contains 17.5 mg of gentamicin per ml of paste. Gentamicin prevents colonization of microorganisms sensitive to gentamicin in order to protect bone healing. Gentamicin is bactericidal against a wide range of bacterial infections, mainly Gram-positive bacteria like Staphylococcus, but also many Gram negative bacteria including Pseudomonas, Proteus, Serratia, which are also found in chronic osteomyelitis.

Cerament-G is indicated for use as a bone void filler material in a surgical procedure where there is a risk of bacterial contamination. It is therefore indicated to fill gaps and bone deficits in the skeleton that do not involve the stability of the bone structure, especially in the limbs. These bone defects may appear spontaneously or have been created surgically or following trauma to the bone, have been identified during primary surgery or surgical revision. It can also be bone defects identified around rigid implant devices.

Cerament-G will be used in this study for the management of patients with chronic osteomyelitis in the innovative strategy, as an adjunct during surgery usually performed by corticotomy. It will be used in accordance with the indications of the CE marking and its instructions for use. The volume of Cerament-G used during the intervention will be planned according to the volume of the cavity to be filled; it is on average 15 ml and cannot exceed the maximum volume corresponding to a gentamicin concentration of 6 mg/kg of patient weight. The volume to be administered will be discussed at a meeting of the eligibility validation committee, which will propose an optimal volume given the characteristics of the patient. In the event of a very large defect, the use of part of the volume of Cerament-G to form beads (or pellets) may be proposed, which would contribute to the best possible filling while limiting the total volume to be used.

#### 2.4.2. Participant timeline

The first inclusion took place on October 14, 2021. Total inclusion period is expected to be 3 years and the duration of participation of each patient will be 25 months. Schedule for enrolment, interventions and assessments are summarized in Table 1.

**Table 1:** Schedule for enrolment, interventions and assessments.

Moment actions	STEPS							
	V1 Inclusion	V2 Hospitalization / Corticotomy	V3 Follow-up consultation	V4 Follow-up consultation	V5 Follow-up consultation	V6 Follow-up consultation	V7 Follow-up consultation	V8 End of study
	D-60 à D-2	D0	W + 4/W + 6	M3 +/− 15 d	M6 +/− 15 d	M12 +/− 15 d	M18 +/− 30 d	M24 +/− 30 d
Informed consent	X							
Randomization	X							
Antecedents	X							
Urine pregnancy test (only for women of childbearing age)	X							
Physical examination	X		X	X	X	X	X	X
Determination of calcium, phosphorus, albumin, urea, and creatinine (serum creatinine and creatinine clearance / measurement of GFR)	X	X	X	X				
EQ-5D-5L^a^	X		X	X	X	X	X	X
Resource consumption			X	X	X	X	X	X
Professional and informal help	X		X	X	X	X	X	X
Micro-costing^b^		X						
Corticotomy / fracture complication		X	X	X	X	X	X	X
Acute renal failure		X	X	X				
Healing and bone remodeling / consolidation (standard radiology)	X					X		
Infectious recurrence		X	X	X	X	X	X	X
Surgical revision^c^			X	X	X	X	X	X
Amputation			X	X	X	X	X	X
Adverse events	X	X	X	X	X	X	X	X

a In the event of surgical revision, an EQ-5D will be completed by the patient closest to the event.

b Micro-costing will only be carried out for patients included in the innovative strategy, with the use of Cerament-G.

c In the event of surgical revision, the schedule begins again in accordance with post-surgical follow-up, until the normal end of the study, i.e., 24 months after the first corticotomy, is reached.

Patients with chronic osteomyelitis of the long bones (stage III of the Cierny-Mader classification) will be identified during the multidisciplinary meeting carried out within the participating CRIOAc.

The patient, if eligible, is informed of the completion of the study during a visit carried out with a view to planning his surgical intervention (pre-surgical visit) or during a dedicated visit, by the investigator. A written information leaflet is given to the patient.

The pre-surgical visit and the necessary examinations are carried out in accordance with usual practices. Hospitalization for the corticotomy is scheduled in accordance with the usual management no later than 2 months after the inclusion visit in order to limit the risk of canceling the surgery and therefore of the patient leaving the study prematurely.

The eligibility validation committee will meet to validate the patient’s inclusion in the study, based on available clinical data and imaging. If the committee confirms the patient’s eligibility, randomization is carried out and the patient assigned to one of the two study groups: the standard of care (usual medico-surgical management) or the innovative strategy (medico-surgical management with the use of Cerament-G during corticotomy surgery). If the committee invalidates the patient’s eligibility, the patient is withdrawn from the study.

Hospitalization for corticotomy and follow-up visits are carried out in accordance with the usual practices of each center.

If the investigator decides to perform a surgical revision before the 24-month visit after the initial surgery, the patient will not be withdrawn from the study but will be provided with follow-up until the normal end of the study, i.e., 24 months after the initial intervention.

In the event of an infectious recurrence or a complication requiring a new surgical intervention, the same follow-up schedule is set up after the surgery to replace the previous one and within the limit of the duration of the study (total follow-up of 24 months after the first intervention).

The end-of-study visit is the one scheduled 24 months (+/− 30 days) after the first corticotomy surgery. In the event of an infectious recurrence or complication requiring surgery and modifying the follow-up schedule, an end-of-study visit will be scheduled 24 months after the initial surgery specifically for the needs of the study.

### 2.5. Primary outcome measure

The primary outcome is the incremental cost-utility ratio estimated at 24 months between two treatments strategies for chronic long bone osteomyelitis.

### 2.6. Secondary outcome measure

Secondary outcomes are classified as efficacy secondary outcome measures or economic secondary outcome measures.

#### 2.6.1. Efficacy secondary outcome

- Proportion of patients with at least one recurrence of infection on the studied bone at 24 months (confirmed by the event validation committee) and time to recurrence;
- Number and types of intraoperative and postoperative complications up to 24 months (including fracture) according to CTCAE and Clavien-Dindo classifications; and proportion of patients with at least one complication during follow-up;
- Number of repeat surgeries for complication up to 24 months; and proportion of patients who had at least one repeat surgery for complication during follow-up;
- Proportion of patients with amputation of the area containing the bone studied at 24 months;
- Proportion of patients with bone healing and proportion of patients with bone remodeling/consolidation at 12 months, assessed from a standard radiograph (confirmed by the Clinical Event Validation Committee);
- Number and types of serious adverse events (SAEs) attributable to systemic antibiotic therapy following the first intervention; and proportion of patients with at least one SAE attributable to this systemic antibiotic therapy; within 3 months of the first intervention;
- Proportion of patients with acute renal failure within 3 months of the first procedure;
- Number and types of adverse events related to the use of Cerament-G (e.g., hypercalcemia, serous discharge prolonged by scarring, heterotopic ossification, allergies) occurring within 3 months after the first procedure.

#### 2.6.2. Economic secondary outcome measures

- Cost of both strategies estimated at 24 months;
- Cost of a corticotomy procedure using Cerament-G;
- Estimated utility based on the Euroqol EQ-5D questionnaire and on utility scores validated on the French population at inclusion, at S + 4/S + 6, M3, M6, M12, M18 and M24 (usual follow-up visits) as well as after each repeat surgery according to the same schedule;
- Incremental cost-effectiveness ratio estimated at 24 months with no recurrence of infection as efficacy outcome measure;
- Budget impact analysis carried out from the perspective of the French Health Insurance.

### 2.7. Health economic evaluation

#### 2.7.1. Main characteristics of the evaluation

Given the working hypotheses, and in accordance with the recommendations of the French National Health Authority (14), the cost-utility study will be conducted from the collective perspective.

A time horizon of 24 months after the initial surgery will be used, which allows to take into account most important costs associated with the surgery and its consequences. Costs and results will be discounted using a 2.5% rate.

#### 2.7.2. Cost evaluation

The cost assessment will take into account hospitalizations (complete or day) in medicine, surgery, obstetrics, follow-up and rehabilitation care and at home, biological and radiological examinations, consultations of a general practitioner or a specialist (infectious disease specialist, orthopedic surgeon, etc.), nursing and physiotherapy care, consumption of drugs (analgesics and antibiotics), transport of patients, professional home help and informal care.

These costs will be valued using production cost or, if not possible, according to the corresponding tariff. The replacement cost approach, also known as “proxy good method,” will be used to value informal caregiver time. This approach assumes that any informal helping activity can be done by a worker in the market. According to this principle, each activity is valued according to what it costs in the labor market. One hour of help will therefore be valued at the average hourly wage of a professional help (15, 16).

For patients’ hospital stays, data will be collected from medical information departments of each participating center.

The number of subjects included in the study is relatively small and these patients are provided with follow-up regularly in hospital. In addition, the patient will need to enlist the cooperation of his/her caregiver to complete the data on informal care, based on an adaptation of existing questionnaires (17, 18). For these reasons, a booklet will be given to the patient on discharge from hospital to collect the consumption of outpatient resources as well as the data necessary for the evaluation of professional and informal help.

Corticotomy surgery incorporating the use of Cerament-G is an innovative procedure whose production cost has never been evaluated and for which no tariff is available since it is not currently reimbursed by the French Health Insurance: a micro-costing analysis will therefore be performed. Since the cost of corticotomy surgery without the use of Cerament-G is known, we will focus on the additional resources consumed when using Cerament-G, i.e., the medical device, the additional duration of use of the operating room, the staff time required for the preparation and injection of Cerament-G. The data needed to estimate these costs will be collected in the study’s case report form. Block occupancy and staff time will be valued from cost accounting, and the medical device from its non-negotiated acquisition price.

#### 2.7.3. Measurement of the result in the medico-economic evaluation (primary outcome measure)

The outcome criterion for the main medico-economic evaluation will be the number of Quality Adjusted Life Years (QALY).

In accordance with the recommendations (14), QALYs will be estimated from the responses to the Euroqol EQ-5D questionnaire. We will use the 5-Level version with the preference scores validated on the French population (19).

We hypothesize that the EQ-5D questionnaire will be sufficiently sensitive to variations in quality of life. However, as a precaution a secondary cost-effectiveness study will be conducted, with freedom from recurrence of infection at 24 months as the clinical outcome.

#### 2.7.4. Presentation and interpretation of results

Once the cost and the number of average QALYs per patient have been calculated for each of the two strategies studied, the results will be represented in a cost-utility plan. This graph, which represents the cost differential on the x-axis and the utility differential on the y-axis, shows whether one of the two strategies is dominated. If the innovative strategy is less costly and more effective than the standard of care, it will be efficient. Conversely, if it is more costly and less effective, we can conclude that it is not efficient. In all other cases, we will have to relate the two dimensions of cost and utility by estimating the incremental cost-utility ratio.

If the innovative strategy is less costly and less efficient, the ratio will provide information on the reduction in efficiency that must be made in order to reduce costs. If the innovative strategy is more costly and more efficient, the ratio will provide with information on the additional cost generated by the innovative strategy compared to the standard of care per QALY gained. In the latter case, an acceptability curve will be constructed, representing the probability that the innovative strategy is cost-effective compared to the standard of care, as a function of the collectivity’s willingness to pay (in other words of the value assigned by the collectivity to a QALY). It will make it possible to visualize the intervention that maximizes the net benefit according to the accepted thresholds of acceptability.

#### 2.7.5. Budget impact analysis

If the innovative strategy proves to be efficient, a budget impact analysis (BIA) is planned. In France, when a medical device enters into common law, the results of the BIA, like those of the medico-economic evaluation, can be used by the Economic Committee for Health Products when negotiating the price with the manufacturer.

The objective of the BIA in the CONVICTION study will be to estimate the impact of the introduction and dissemination in the French health system of the innovative strategy in the treatment of chronic osteomyelitis of long bone. The BIA will therefore provide information to decision-makers on the financial sustainability of this innovation, which will complement the information on efficiency provided by the medico-economic evaluation.

In accordance with the recommendations of the French National Authority for Health (20), the BIA will be carried out from the perspective of French Health Insurance. A 5-year time horizon will make it possible to take into account the gradual substitution of the standard of care by the innovative strategy using Cerament-G.

### 2.8. Data collection

The study data will be collected in an electronic case report form (eCRF). This eCRF, specific to the study, will be developed by a data manager from the Hospices Civils de Lyon using Ennov Clinical® 7.5.720 software. This software complies with the recommendations of the FDA on computerized systems for the management of clinical trials (Guidance for Computerized Systems Used in Clinical Trials) as well as the recommendations of the FDA on the electronic signature (21CFR part 11).

The CRF will only include the data necessary for carrying out the protocol and for scientific publication. The other patient data necessary for their follow-up outside of this study will be collected in the patient’s medical file.

### 2.9. Safety and adverse events monitoring

All adverse events occurring within the study, from inclusion, will be recorded in the eCRF and will be graded according to the Clavien-Dindo scale and/or the Common Terminology Criteria for Adverse Events (CTCAE). The investigator will determine causality in relation to the study, the procedure and/or the use of the device. All adverse events with great intensity, life threatening and death (i.e., ≥3 on the CTCAE and ≥ 3a on the Clavien-Dindo scale) will be considered serious and notified to the sponsor.

As Cerament-G is CE-marked and used in accordance with the manufacturer’s Instruction For Use, all incidents and risk of incidents will be notified to the sponsor and to the French Agency for the Safety of Health Products *via* the local material surveillance correspondent of the participating center.

### 2.10. Statistical considerations

#### 2.10.1. Sample size calculation

The sample size calculation has been based on the most relevant clinical efficacy outcome measure, which is the proportion of patients with at least one recurrence of infection at 24 months.

Available data show rates of 4 to 5% when using Cerament-G, for durations between 5 and 21 months (10, 11); as the follow-up is longer in the CONVICTION study (24 months), the hypothesis chosen was a rate of 6%. On the other hand, the proportion of recurrence of infection in the standard of care is estimated at 20% (21). The total number of patients to be randomized in the study will be 200 (100 per group; considering alpha-risk at 0.05, power of 80% in a bilateral situation and approximately 10% of early withdrawal).

The estimate of the number of subjects needed according to medico-economic hypothesis would require numerous hypothesis, leading to uncertainty. Only the cost of Cerament-G and hospitalizations for recurrence of infection, and the impact on the short-term utility of the surgery for recurrence of infection (6% in the innovative strategy vs. 20% in the standard of care), fractures and amputations (5% in both groups) have been taken into account. We hypothesize that the utility is decreased 6 months before revision surgery, 3 months after revision surgery, 9 months after amputation and 3 months after fracture. As no utility data are available in the literature for “before surgery” and “after surgery” conditions, they have been estimated according to expert opinion. As little data is available in the literature for health states comparable to the health states “after fracture” and “after amputation” and for the sake of homogeneity of the measurement, the utility concerning these states has also been estimated by expert opinion. However, these data seem consistent with the closest data available (22).

The utility scores used to value these health conditions are as follows:

- Before surgery = 0.8
- After surgery = 0.49
- After fracture = 0.28
- After amputation = 0.39

Regarding costs, the average cost per patient only for Cerament-G and for the innovative strategy group was estimated at 2,664 € (based on the assumption of using 15 ml of product for a third of patients and 10 ml for the remaining two-thirds). The average cost of a hospital stay was estimated, according to the French study on cost taking into account the different levels of severity, at €13,776 for a leg amputation and at €3,330 for a broken leg. Finally, the cost of a recurrence of infection was estimated, according to an expert, at approximatively €10,000.

The average cost difference hypothesis was €1,144, with a standard deviation at €1,000, and the average difference of QALY hypothesis was 0.04, with a standard deviation at 0.03. For a maximum willingness to pay of €50,000, a correlation coefficient of −0.03, an alpha risk of 5% and a beta risk of 20%, with 10% of early withdrawal, the number of subjects needed was estimated at 196 (98 per group). As this estimate is conservative, the estimated number of subjects needed corresponds to a high value.

The randomization of 200 patients in the study will therefore validate both the efficiency and the clinical efficacy hypotheses. Finally, considering that 10% of patients whose inclusion will be invalidated by the Patient Eligibility Validation Committee, a total of 220 patients will be included.

#### 2.10.2. Statistical analysis

The characteristics of the overall population and the two groups will be described. The analysis will be carried out on an intention-to-treat basis. Usual descriptive statistics will be presented for costs and QALYs. Depending on normality test results, mean costs will be compared across groups using Student’s t-test or non-parametric bootstrap. The 95% confidence interval for incremental cost-utility ratio will be computed using Fieller’s method or the bootstrap method. Deterministic sensitivity analyses will be performed, the results of which will be presented in a tornado diagram.

The statistical analysis to estimate the treatment effect on clinical outcomes will be based on the following methods: a linear regression model adjusted for stratification criteria for continuous variables; a logistic regression model adjusted for stratification criteria for dichotomous variables; and a Cox model adjusted for stratification criteria for survival data.

## 3. Discussion

Despite the progress made in both antibiotics and surgical treatment, the management of chronic osteomyelitis of long bone could be improved. Probability of failure of treatment is around 20% and that rate has unfortunately remained stable for more than 20 years. As it is mainly a local disease, the only way to improve the prognosis is probably to act locally, to increase the probability of pathogen eradication, and to prevent superinfection and fracture.

One historical way to better target the pathogen responsible for infection is to deliver conventional local antibiotics during surgery, but all traditional antibiotics usable for injections are available in liquid form after reconstitution. Some surgeons historically performed bone washing with rifampin or with gentamicin. However, during surgical intramedullar debridement, bone bleeding mechanically pushed away liquid antibiotics that could be used intramedullary. Moreover, the half-life of conventional antibiotics usable intravenously is very short, with no residual active drug 24 h after their reconstitution.

Some authors discussed performance of autologous bone graft to fill the dead space, or the use of antibiotic-impregnated PMMA cement (beads or cementoplasty). However, biomechanically, the bone does not need bone graft in stage III long bone osteomyelitis. Unlike Cerament-G, PMMA cement is not resorbable. Subsequent surgery is required to remove it, and once it is removed, a dead space still remains and must be managed; a biomechanically unnecessary bone graft is often placed at the time of PMMA explantation. Significant complications and morbidity could be associated with this strategy which is recognized as controversial and non-optimal. Finally the Papineau technique is the performance of bone debridement with keeping the wound open and the bone exposed, with performance of serial bone grafting before performing of a skin graft is clearly no longer proposed in contemporary times. Of note, none of these techniques are considered as the standard of care procedure for dead space management, and these practices seem to be largely heterogeneous. In certain situations, surgeons do not have access to Cerament-G or other bone substitutes. Since the superiority of using such devices has not yet been demonstrated, leaving the dead space empty is a current option for stage III long bone osteomyelitis.

Emergent potential local anti-infective treatment, phage or phage-derived therapies could also be future options. Bacteriophages are natural viruses that target specifically a bacterial species. They have the ability to replicate themselves in their host, and can be deliver locally. This lysin, can destroy the bacteria; it also has anti-biofilm properties (23). Phage therapy is a potentially innovative approach for patients with bone and joint infection, but there are major drawbacks: (i) few phages are available at this time, targeting only few bacteria such as *S. aureus* or *P. aeruginosa*; (ii) it is necessary to identify the pathogen(s) responsible for the infection before the surgery, to select active phages after determining phage susceptibility. This is not feasible in patients with chronic osteomyelitis. Phage-derived therapies such as the use of lysins, which are biologically active enzymes, could be of interest for the future (24), as an antibiotic-nanoencapsulated in gel that could be used locally (25).

Finally, the use of a resorbable synthetic bone substitute with local antibiotic eluting capability, such as Cerament-G, having a wide spectrum of activity against the most frequent bacteria involved in the disease, could be the most relevant way to improve the prognosis of chronic osteomyelitis. Indeed, firstly, a bone substitute that has the ability to fill the “dead space” that is caused during surgery, will prevent the stagnation of blood in the intramedullar space. This phenomenon of bone void is a favorable environment for the persistence of the initial pathogen and is probably a factor which promotes the risk of superinfection due to another pathogen that can contaminate the intramedullar cavity during surgery. Secondly, the local delivery of high doses of gentamicin (that is a broad-spectrum bactericidal antibiotic effective against the vast majority of bacteria involved in osteoarticular infections) at prolonged concentrations for several weeks is also a great advantage to treat the current infection (in combination with systemic antibiotics) and prevent superinfection. Finally, a resorbable bone substitute promotes the regeneration of the bone within this space, limiting in theory the risk of fracture during follow-up.

Two prospective studies have shown that Cerament-G was associated with a low number of infectious recurrences (about 5%). However comparative studies, directly evaluating the effectiveness and efficiency of this medical device, are lacking. The CONVICTION study proposes a protocol for a clinical and economic evaluation of an innovative device that could, despite an additional initial cost, lead to fewer costs in the future, while improving the utility of patients. Its results will allow to identify whether the use of Cerament-G during surgery is a cost-effective alternative strategy, compared to the absence of filling the dead space during surgery. This control group strategy was chosen as there is currently no consensus on the management of the patients concerned, and as it seems to represent the current majority practice in France. Furthermore, the randomized design of the study will provide relevant results on the clinical benefit of using such a resorbable synthetic bone substitute with local antibiotic eluting capability for osteomyelitis management.

Study findings could also have an impact on the decision of the funding of this expensive device by French health insurance. To our knowledge, there are no other medico-economic studies published or in progress on this topic.

Osteoarticular infections, including chronic osteomyelitis, represent such a high cost that the cost of even a preventive medical device can be offset by the costs of the infections that the device could prevent (26). More generally, we therefore hope that the results of this study will help to show the clinical and economic impact that a device for preventing osteoarticular infections can have.

The study is done throughout the network of CRIOAc in France, in referral centers for the management of complex infections. This network was established in 2009, and its aim is to treat all French patients by offering specialized medical advice at our centers, located across the country. It would be a key process to perform clinical trials in teams dedicated to the management of bone and joint infections, with experience and skills that have grown since the establishment of these centers (27). Of note, recent epidemiological data show that there is a significant number of long-bone osteomyelitis managed in the network, which leads us to believe that the study is feasible in France (28).

This study has several limitations and particular biases. First, the complete adherence of the investigators is needed, especially if we focus on the training of the surgeons who manipulate the device, and only patients without post-debridement dead space management can be included. Secondly, it was impossible to blind the surgeon, who knows at the time of surgery, if the patient will receive or not the Cerament-G. It is of importance that the surgical debridement of the osteomyelitis of a particular patient has to be the same whether the patient is randomized to one arm or another. Finally, we decided, in particular to collect the data needed to estimate the informal care costs, to use a patient notebook to collect healthcare consumption. This mode of collection can lead to biases if the documents are not correctly completed. However, we closely monitor the filling of the patient notebooks and, if this is not satisfactory, we will consider linking the trial data with the National Health Data System (NHDS) in order to estimate the costs.

## Ethics statement

The studies involving human participants were reviewed and approved by Ethics Committee CPP Sud Méditerranée V on April 06, 2021 (21.03.10.77652) and French National Agency for Medicines and Health Products on May 03, 2021 (2020-A02299-30). The patients/participants provided their written informed consent to participate in this study.

## Author contributions

HS, LH, and TF designed the CONVICTION study protocol and drafted the manuscript. CB and SB contributed to the conception of the study and revised critically the manuscript. All authors contributed to the article and approved the submitted version.

## Funding

This study was supported by a grant from the French Ministry of Health (PRME-19-0007).

## Conflict of interest

The authors declare that the research was conducted in the absence of any commercial or financial relationships that could be construed as a potential conflict of interest.

## Publisher’s note

All claims expressed in this article are solely those of the authors and do not necessarily represent those of their affiliated organizations, or those of the publisher, the editors and the reviewers. Any product that may be evaluated in this article, or claim that may be made by its manufacturer, is not guaranteed or endorsed by the publisher.
